# Self‐Healing and Injectable Hydrogel for Matching Skin Flap Regeneration

**DOI:** 10.1002/advs.201801555

**Published:** 2018-11-16

**Authors:** Xiyuan Mao, Ruoyu Cheng, Hongbo Zhang, Jinhong Bae, Liying Cheng, Lu Zhang, Lianfu Deng, Wenguo Cui, Yuguang Zhang, Hélder A. Santos, Xiaoming Sun

**Affiliations:** ^1^ Department of Plastic and Reconstructive Surgery Shanghai Ninth People's Hospital Shanghai JiaoTong University School of Medicine 639 Zhi Zao Ju Road Shanghai 200011 P. R. China; ^2^ Shanghai Key Laboratory for Prevention and Treatment of Bone and Joint Diseases Shanghai Institute of Traumatology and Orthopaedics Ruijin Hospital Shanghai Jiao Tong University School of Medicine 197 Ruijin 2nd Road Shanghai 200025 P. R. China; ^3^ Åbo Akademi University Turku FI‐20520 Finland; ^4^ State Key Laboratory of Molecular Engineering of Polymers Fudan University No. 220 Handan Road Shanghai 200433 China; ^5^ Drug Research Program Division of Pharmaceutical Chemistry and Technology Faculty of Pharmacy University of Helsinki Helsinki FI‐00014 Finland; ^6^ Helsinki Institute of Life Science (HiLIFE) University of Helsinki Helsinki FI‐00014 Finland

**Keywords:** flap regeneration, liposomes, mangiferin, multifunctional hydrogels, polyethylene glycol

## Abstract

The fabrication of highly biocompatible hydrogels with multiple unique healing abilities for the whole healing process, for example, multifunctional hydrogels with injectable, degradation, antibacterial, antihypoxic, and wound healing–promoting properties that match the dynamic healing process of skin flap regeneration, is currently a research challenge. Here, a multifunctional and dynamic coordinative polyethylene glycol (PEG) hydrogel with mangiferin liposomes (MF‐Lip@PEG) is developed for clinical applications through Ag–S coordination of four‐arm‐PEG‐SH and Ag^+^. Compared to MF‐PEG, MF‐Lip@PEG exhibits self‐healing properties, lower swelling percentages, and a longer endurance period. Moreover, the hydrogel exhibits excellent drug dispersibility and release characteristics for slow and persistent drug delivery. In vitro studies show that the hydrogel is biocompatible and nontoxic to cells, and exerts an outstanding neovascularization‐promoting effect. The MF‐Lip@PEG also exhibits a strong cytoprotective effect against hypoxia‐induced apoptosis through regulation of the Bax/Bcl‐2/caspase‐3 pathway. In a random skin flap animal model, the MF‐Lip@PEG is injectable and convenient to deliver into the skin flap, providing excellent anti‐inflammation, anti‐infection, and proneovascularization effects and significantly reducing the skin flap necrosis rate. In general, the MF‐Lip@PEG possesses outstanding multifunctionality for the dynamic healing process of skin flap regeneration.

Hydrogels are biomaterials widely used for tissue repair in tissue engineering. With their biocompatibility and tunable architecture, hydrogels can simulate the physical structure of the extracellular matrix to promote cell proliferation and tissue regeneration.^1^, ^2^, ^3^ Hydrogels are able to encapsulate and deliver different drugs for various kinds of tissue repair processes, for example, flap regeneration, skin healing, and bone reconstruction.^4^, ^5^, ^6^ Injured tissue repair is a dynamic and complex process. During the early stage of skin flap recovery, tissue suffers from damaged homeostasis and cell apoptosis caused by inflammation, ischemia, and hypoxia; then, in the proliferation period, the cells proliferate rapidly after endothelial cells exhibit adequate vascularization, while fibroblasts and mesenchymal stem cells help with healing and regeneration. Finally, during the remodeling period, the tissue undergoes structure and vascular network remodeling and maturing, which take months or years to for the tissue to eventually regain its normal function.^7^, ^8^ However, little research has been done to optimize hydrogels' drug releasing and degradation property to match with each dynamic healing process. Therefore, multifunctionalized hydrogels that could synchronize with tissue physiological healing process would have significant potential in bioengineering applications.

For example, in the early stage of random skin flap reconstruction, deprivation of adequate blood supply causes flap hypoxia, ischemia/reperfusion, and inflammation, especially in the distal part. The unfavorable hypoxic environment hinders flap rapid regeneration during the early stages. To address this problem, previous studies mainly focus on using hydrogels as various drugs and cytokine delivery systems to reduce distal end necrosis in the skin flap. However, using cytokines to stimulate angiogenesis does not protect cells from hypoxia, high concentrations of cytokines and drugs can cause side effects and even impair healing.^9^, ^10^, ^11^ Moreover, in these studies, the hydrogels only act as physical supports to deliver cargos, and thus, the healing effect of these hydrogels is not an area of concern.^12^

Hydrogels can be modified to improve their performance in tissue engineering, including cell proliferation, new microvascular network formation, and tissue extracellular matrix structure construction.^13^, ^14^ Although many studies have focused on the chemical and physical internal structure of hydrogels, the healing improvement capacity of hydrogels themselves has received little attention.^15^ Hydrogels delivered to the wound area during the tissue healing process can directly contact and interact with the wounded cells. The hydrogel's drug releasing and degradation property could be modified to match with the dynamic healing process.^16^ Herein, we investigate the biological repair and healing effect of hydrogels with the goal to uncover its role and mechanisms in tissue repair and regeneration.

In this work, we crosslinked four‐armed polyethylene glycol (PEG) with Ag^+^ by Ag–S coordination to form a dynamic coordinative and injectable PEG‐Ag hydrogel that was loaded with mangiferin liposomes (MF‐Lip) to promote skin flap survival rate through the combination of the cytoprotective effect of mangiferin and sustained anti‐infection and accelerated wound healing activity of Ag^+^ under hypoxic conditions.^17^ The dynamic coordinative PEG hydrogel constructed by Ag–S coordination exhibited outstanding injectable, self‐healing, degradable, and anti‐infection properties. In addition, mangiferin (MF), a naturally occurring glucosyl xanthone commonly found in mango and papaya, has been suggested to possess various pharmacological activities, such as antioxidant, anti‐inflammatory, antiapoptotic, and immunomodulation effects.^18^, ^19^, ^20^, ^21^ Herein, we fabricated a hydrogel that is able to locally release Ag^+^ and MF and that exhibit anti‐infection, cytoprotective, and angiogenesis activity under hypoxic conditions. The hydrogel delivery system slowly degrades with the skin flap healing process while guaranteeing enough mangiferin concentration in situ to promote skin flap regeneration. With the combination of cytoprotective and angiogenesis of mangiferin and the anti‐infection and healing promotion ability of Ag^+^, the hydrogel delivery system is expected to match the renewal of random skin flap regeneration progress and promote skin flap survival. (**Scheme** **1**).

**Scheme 1:**
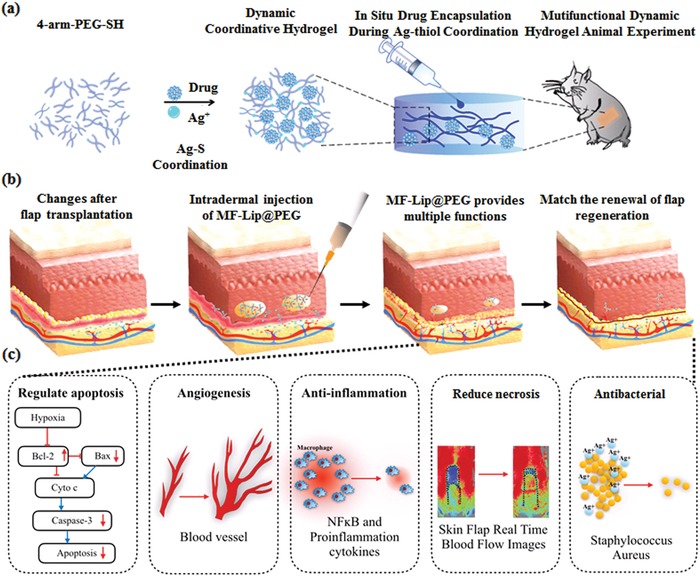
Schematic illustrations of MF‐Lip@PEG matching the renewal of random skin flap regeneration. a) Four‐arm‐PEG‐SH, MF and Ag^+^ constructed by Ag–S coordination form the dynamic coordinative hydrogel. b) MF‐Lip@PEG was injected into the derma, and the MF‐Lip was encapsulated in situ during the Ag–thiol coordination. The hydrogel continued to release MF‐Lip and Ag^+^ while slowly degrading with the flap healing process. c) The MF‐Lip@PEG promote skin flap survival rate through the combined cytoprotective, neovascularization‐promoting, anti‐inflammation, and antibacterial effects and the reduction in skin flap necrosis rate under hypoxic conditions, matching the renewal of flap regeneration.

MF was loaded in the lipid bilayer of liposomes, which consists of phosphatidylcholine and cholesterol (Figure S1, Supporting Information). The morphology of the MF‐Lip was investigated by transmission electron microscopy (TEM) (Figure S2, Supporting Information) and scanning electron microscopy (SEM) (Figure S3, Supporting Information). According to these images, the nanoparticles showed a bilayer structure; specifically, the darker parts were the inner water phase of the liposomes, and the lighter parts indicated the lipid bilayer. For the surface properties, lyophilized liposome powder with a round smooth surface was observed, as shown in Figure S3 in the Supporting Information. To further characterize the MF liposomes, their size distribution and polydispersity index (PDI) were investigated (Figure S4, Supporting Information): the average size of the MF‐Lip was 142.7 ± 2.4 nm, and the PDI was 0.140 ± 0.023. Moreover, the zeta potential distribution is shown in Figure S5 in the Supporting Information, and the mean zeta potential was −8.57 ± 1.11 mV. The entrapment efficiency of these liposomes was 66.7 ± 3.2% and the drug release in vitro lasted for almost 24 h, with the cumulative percent release reaching over 75% (Figure S6, Supporting Information).

To explore the process of gelation (**Figure** **1**a,b), it was observed that before mixing the solution of four‐arm PEG‐thiol with AgNO_3_, the solution of the materials had excellent flowability. After mixing these two solutions, a composite gel containing MF‐Lip was formed. Furthermore, the injection capability of the gel was confirmed, as shown in Figure 1c. The rhodamine‐dyed MF‐Lip@PEG was continuously injected though the syringe with a 0.5 mm needle. The hydrogels were also injected into a centrifuge tube filled with deionized (DI) water to prove that they can not only be injected but also maintain their shape in solution, as shown in Figure 1d−f. The surface structure of the hydrogel was observed by SEM at 1 h, as shown in Figure S7 in the Supporting Information. At low magnification, there were no significant differences among these diverse groups from PEG to MF‐Lip40@PEG (Figure S7, Supporting Information). In contrast, some nanoparticles were observed on the surface of the MF‐Lip@PEG with different concentrations of MF‐Lip under high magnification; particularly, increasing amounts of MF‐Lip led to an increase in the number of nanoparticles on the surface of the composite hydrogels. Furthermore, the magnified SEM images also showed a successful combination between the MF‐Lip and the PEG hydrogel.

**Figure 1:**
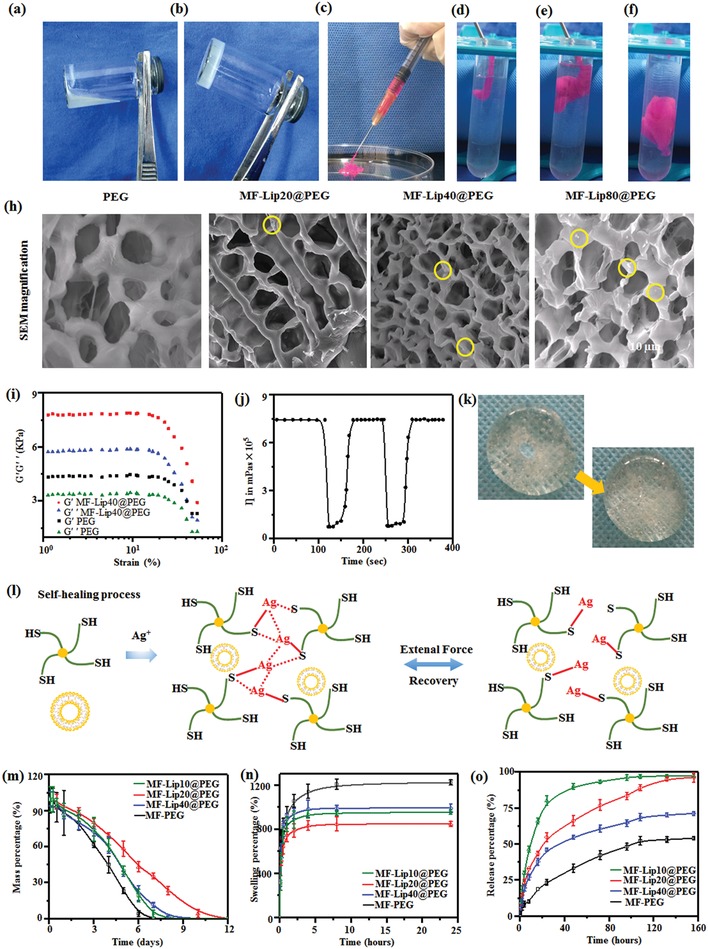
Related properties of MF‐Lip, morphological examination of the hydrogels, and characterization of the hydrogels. a) Solution of four‐arm PEG‐thiol with MF‐Lip. b) The MF‐Lip@PEG hydrogel. c) Photograph of the injectable hydrogel. d–f) Images of a hydrogel injected into DI water. h) Magnified SEM results (liposomes are marked by yellow circles). i) Strain sweep measurements of the storage moduli (*G*' denotes the elastic modulus, and *G*” denotes the loss modulus), measured in kPa, and their effect on the strain of the hydrogels (%), which was used to determine their tensile strength. j) Measure of viscosity parameters in relation to time in seconds. The strain shearing rate alternated between 0.05% strain for 100 s and 500% strain for 50 s. k) The process of self‐healing. l) The mechanism of the self‐healing ability and injectability. m) Degradation. n) Swelling percentage. o) In vitro drug release.

Before considering its drug‐carrying capabilities, the hydrogel has to be able in exhibit self‐healing properties and endure external strain. As shown in Figure 1i, both the elastic modulus (*G*') and loss modulus (*G*”) could withstand an increase in strain of ≈50%. Furthermore, upon examination of the recoverability by a rheometer, it was observed that before applying high shear rate, the hydrogel was able to maintain a colloidal state while keeping the viscosity at ≈7.5 × 10^5^ Pas; then, when a high shear rate was applied, the hydrogel's structure was destroyed, and the viscosity of the hydrogel dropped to almost 0.7 × 10^5^ Pas. However, a few seconds after the high shear rate was removed, the hydrogel recovered to a state similar to the one prior to being subjected to the external shear rate (Figure 1j). From a macro view, the same process was also observed in photographs (Figure 1k), where a hole was created in the center of the hydrogel after ≈10 min, followed by recovery from the damage. The mechanism of the process is described in Figure l. During the mixing of four‐arm PEG‐thiol with AgNO_3_, the covalent bonds formed between S and Ag, as well as the simultaneous formation of interactions between Ag and Ag, transformed the solution to the gel.^22^, ^23^ When an intense external shear rate was applied, these bonds were destroyed, and when these external forces were removed, the network regenerated. Therefore, we predicted at this point that the hydrogels were capable of recovery after injection.

Regarding implantation, a hydrogel should have the capability to not only maintain its integral construction during the treatment process, but also undergo degradation. As shown in Figure 1m, MF‐Lip40@PEG showed the longest endurance period for 12 d compared to 9 d for both MF‐Lip20@PEG and MF‐Lip10@PEG, while the shortest time was 7 d for PEG. Regarding the swelling percentages, PEG exhibited the highest value (almost 1200%). In contrast, the swelling percentages in all MF‐Lip@PEG groups were noticeably lower, ranging from ≈900% for both MF‐Lip20@PEG and MF‐Lip10@PEG to just 800% for MF‐Lip40@PEG. Increasing amounts of MF‐Lip were added to the hydrogel system, leading to a denser network structure in the composite hydrogels, and resulting in lower swelling percentages and longer endurance periods of the MF‐Lip40@PEG, because the dense network could serve as a barrier to the movement of water molecules.^24^, ^25^

MF is a hydrophobic drug and tends to aggregate upon direct blending with a solution of four‐arm PEG‐thiol and AgNO_3_. Thus, MF‐PEG produced by this method showed poor drug dispersion, and the release percentage of MF from PEG‐MF was the lowest (≈50%). Almost half of the loaded MF was not released from the MF‐PEG because of severe aggregation of MF in the hydrogel matrix. In contrast, the liposomes were able to carry MF in their bilayer, leading to excellent dispersibility of the drug in the composite hydrogel networks. As a result, the cumulative release percentages of MF were significantly improved from 50% in MF‐PEG to ≈95% in the three MF‐Lip@PEG groups. Specifically, these drugs would be released in several methods in vitro. First, these drugs would be released from the bilayers with the leakage of liposome, and second, these drug‐loaded liposomes would be released from the composite hydrogel, and then these drugs could be detected in the release medium. In particular, the MF‐Lip40@PEG exhibited more favorable release characteristics than those of the other two MF‐Lip@PEG groups, because the dense network is capable of making contributions to sustain the in vitro drug release.^26^, ^27^

The beneficial effects of MF‐Lip@PEG were investigated in human endothelial vein cells (HUVECs), and the angiogenic activity of the liposome was studied in vitro. HUVECs were treated with different concentrations of MF‐Lip@PEG leaching liquor for 7 d, and the protein expression levels of vascular endothelial growth factor (VEGF) and basic fibroblast growth factor (bFGF) were identified by western blot analysis. As shown in **Figure** **2**, at concentrations below 40 × 10^−6^ m, the MF‐Lip40@PEG could significantly increase the expression level of VEGF and bFGF in a dose‐dependent manner. Moreover, the expression level of VEGF and bFGF in the groups treated with MF‐Lip10@PEG, MF‐Lip20@PEG, and MF‐Lip40@PEG was significantly higher than those in the control group. However, the promotion effect of MF‐Lip60@PEG was dramatically decreased, similar to the results observed for MF's effect on cell viability (Figures S8 and S9, Supporting Information). Therefore, the concentrations of MF‐Lip10@PEG, MF‐Lip20@PEG, and MF‐Lip40@PEG were chosen for further studies.

**Figure 2:**
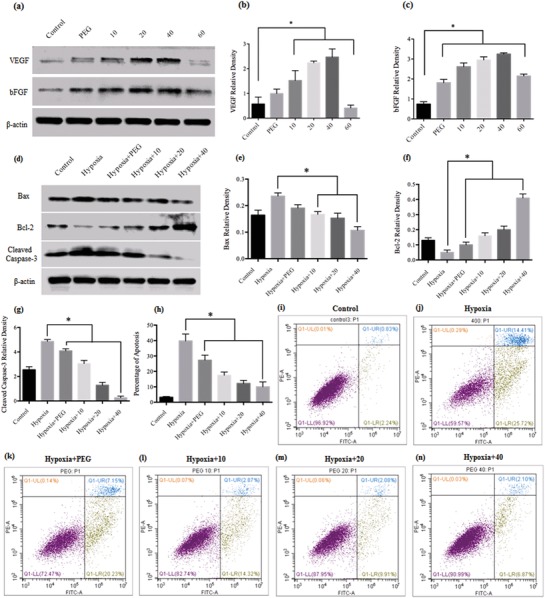
The effects of MF‐Lip@PEG on HUVEC angiogenesis ability and the protective effect of MF‐Lip@PEG on 400 × 10^−6^ m CoCl_2_‐damaged HUVECs. a) Western blotting data showing the levels of angiogenesis‐related growth factors with different MF treatments after 7 d. Western blotting data of the levels of b) VEGF and c) bFGF with different MF‐Lip@PEG treatments after 7 d. d) Western blotting data showing the levels of apoptosis pathway‐related protein expression with different treatments after 24 h. Western blotting data of the levels of e) Bax, f) Bcl‐2, and g) cleaved‐caspase 3 with different MF‐Lip@PEG treatments 24 h. h–n) Flow cytometric analysis of cell apoptosis after 24 h of treatment with 400 × 10^−6^ m CoCl_2_ and PEG or different concentrations of MF‐Lip@PEG. Hypoxia: cells were treated with 400 × 10^−6^ m CoCl_2_ to stimulate hypoxia‐induced damage and were not treated with MF‐Lip@PEG; PEG: PEG leaching liquor; 10: MF‐Lip10@PEG leaching liquor; 20: MF‐Lip20@PEG leaching liquor; 40: MF‐Lip40@PEG leaching liquor; 60: MF‐Lip60@PEG leaching liquor. *p < 0.05.

Endothelial cells are essential cells for blood vessels, and they play an important role in blood vessel formation.^28^ The vascular endothelial cells can secrete a variety of vasoactive substances via autocrine and paracrine mechanisms.^29^ A study reported that MF could increase endothelial cell migration in a dose‐dependent manner, indicating that MF has beneficial effects on the formation of new blood vessels.^30^ Angiogenesis is very important in the early stage of healing. VEGF and bFGF are growth factors that play a central role in angiogenesis by promoting neovascularization and endothelial cell proliferation. A lack of these factors can result in abnormal vessel formation.^31^, ^32^ In the present study, we found that following treatment with MF‐Lip@PEG leaching liquor for 7 d, the expression levels of VEGF and bFGF in HUVECs increased significantly in a dose‐dependent manner at concentrations below 40 × 10^−6^ m (Figure 2b,c). The present results indicate that the MF‐Lip@PEG leaching liquor could promote the cells' angiogenesis ability.

As a result of the MF‐Lip's antiapoptotic effect, the MF‐Lip@PEG was expected to have cell protection ability under hypoxic conditions. To investigate this effect, we stimulated a hypoxic environment in vitro. A hypoxic microenvironment for HUVECs was induced with 400 × 10^−6^ m CoCl_2_ (Figures S10 and S11, Supporting Information). The HUVECs were further treated with PEG, MF‐Lip10@PEG, MF‐Lip20@PEG, or MF‐Lip40@PEG leaching liquor, and cell apoptosis was assessed by flow cytometry. In the hypoxia group, the cell apoptosis rate was 39.8 ± 4.5%. Significantly decreased cell apoptosis rates were observed in the MF‐Lip@PEG leaching liquor‐treated groups. Additionally, the apoptosis rate in the MF‐Lip10@PEG group was 17.5 ± 2.1%, whereas that in the MF‐Lip20@PEG group was 12.2 ± 2.0% and that in the MF‐Lip40@PEG group was 10.0 ± 3.0%. In the MF‐Lip@PEG leaching liquor groups, CoCl_2_‐induced cell apoptosis was significantly inhibited in a dose‐dependent manner. Significantly decreased cell apoptosis was observed in the MF‐Lip@PEG‐treated groups compared to the PEG‐only group and the hypoxia group (Figure 2h−n), indicating that MF‐Lip@PEG leaching liquor could ameliorate apoptosis in the hypoxia‐induced cells. It is worth noting that the apoptosis rate in the PEG group was 27.3 ± 3.2%, which was also significantly lower than that in the hypoxia group, therefore suggesting that the PEG leaching liquor can also significantly inhibit 400 × 10^−6^ m CoCl_2_‐induced cell apoptosis.

It was observed that the MF‐Lip@PEG was capable of attenuating CoCl_2_‐induced cell apoptosis in HUVECs. We next sought to determine the underlying mechanisms by examining protein expression of the apoptosis‐related proteins cleaved‐caspase‐3 and Bax/Bcl‐2. Treatment with MF‐Lip@PEG leaching liquor significantly reduced the cells' cleaved‐caspase‐3 expression levels in a dose‐dependent manner (Figures 2d,g). Similarly, the MF‐Lip@PEG leaching liquor significantly reversed the CoCl_2‐_induced expression of Bax/Bcl‐2 (Figure 2d−f). The Bax/Bcl‐2/caspase‐3 pathway is very important in the process of apoptosis.^33^ Bax is a proapoptotic protein that is polymerized after apoptotic provocation and generates pores in the mitochondrial membrane, subsequently inducing cell apoptosis.^34^ Bcl‐2 is a major antiapoptotic protein and can regulate apoptosis by binding to Bax and inhibit its apoptotic effect.^35^ Therefore, the Bcl‐2/Bax ratio is a crucial factor in cell survival. Members of the caspase family are essential proteins in cell apoptosis, among which caspase‐3 is crucial effector during the apoptotic process.^36^ The current study indicates that the MF‐Lip@PEG can protect hypoxia‐damaged HUVECs through regulation of the Bax/Bcl‐2/caspase‐3 pathway. Moreover, it was observed that PEG leaching liquor also protected the HUVECs from apoptosis and upregulated the Bcl‐2/Bax ratio, suggesting that the PEG leaching liquor alone exerted a protective effect on the hypoxia‐damaged HUVECs cells.

To further demonstrate the persistent skin flap regeneration‐promoting capability of the hydrogel in vivo, the effect of the hydrogel on a random‐pattern skin flap rat model was investigated. An ≈1.5 × 5 cm random skin flap was elevated on the dorsal side of the rats (**Figure** **3**a), and the hydrogel was intradermally injected into the skin flap before the flap was put back in its original position (Figure 3b,c). Different concentrations of MF directly blended in the hydrogel for the skin flap treatment were also investigated (Figure S12, Supporting Information). At 7 d after treatment, the rats were anesthetized, and the skin flap survival rates were assessed using a moorFLPI (Moor instruments, UK) to detect the real‐time flap blood flow. The contrast images obtained were color‐coded to correlate with blood flow of the flap (Figure 3e). The average necrosis rate in the control groups was ≈38.6 ± 1.25% (*n* = 6), while the average necrosis rate in the 40 × 10^−6^ m MF‐Lip group was 23 ± 0.1% (*n* = 6) and the average necrosis rate in the PEG group was 28.4 ± 1.4% (*n* = 6). Lower skin flap necrosis rates were observed in flaps treated with the MF‐Lip@PEG. For the MF‐Lip10@PEG group, the average necrosis rate was 25.4 ± 2.3% (*n* = 6); for the MF‐Lip20@PEG group, the average necrosis rate was 20.8 ± 0.8% (*n* = 6); and for the MF‐Lip40@PEG group, the average necrosis rate was 12.8 ± 1.2% (*n* = 6). It was observed that, without loading of the MF‐Lip into the hydrogel, PEG alone could significantly increase the skin flap survival rate. Additionally, the skin flap survival rates further increased with the MF‐Lip@PEG in a dose‐dependent manner.

**Figure 3:**
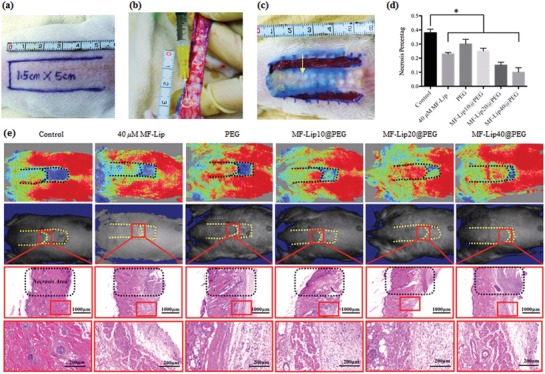
The skin flap survival rates after treatment. a) A random skin flap animal model, in which the flap was 1.5 cm (width) × 5 cm (length) on the rats' dorsal side with the pedicle at the tail end. b,c) After flap elevation, 0.3 mL of hydrogel solution was intradermally injected into the flap skin, and the hydrogel was injected evenly into the flap at 20 dots for each flap (yellow arrow). d) Flap necrosis area percentages of different groups; e, first row): the laser speckle contrast imaging captured real‐time blood flow images of different groups. e, second row): photos of skin flaps in different groups; e, third row), H&E staining of the necrosis and survival junction area of the skin flaps in different groups. e, last row), Magnified view of the survival area in different groups. *p < 0.05.

Skin flap angiogenesis was studied to further investigate the neovascularization effect of the hydrogel on the skin flap. CD31 immunohistochemical staining was performed in order to study the skin flap neovascularization. Cross‐sections of the flap revealed that PEG and the MF‐Lip@PEG significantly increased the densities of the flap microvessels. After 7 d, massive microvessels were seen in the PEG‐treated groups (**Figure** **4**a, the red dots marked the microvessels). The average microvessel densities were 20.2 ± 12.0 microvessels/spot in the control group; 45.0 ± 5.0 microvessels/spot in the 40 × 10^−6^ m MF‐Lip group; 70.6 ± 5.0 microvessels/spot in the PEG group; 42.0 ± 3.2 microvessels/spot in the MF‐Lip10@PEG group; 50.0 ± 5.5 microvessels/spot in the MF‐Lip20@PEG group; and 55.7 ± 4.2 microvessels/spot in the MF‐Lip40@PEG group. The CD31 staining was significantly increased in the treatment groups, and there was no difference between the 40 × 10^−6^ m MF‐Lip group and the MF‐Lip40@PEG group (Figure 4c). Immunohistochemical staining of CD31 proved the dose‐dependent pro‐angiogenic effect of the MF‐Lip@PEG. The microvessel density in the PEG group was the highest compared to the other groups (Figure 4). This was probably caused by the mild local inflammation or immune response caused by PEG hydrogel delivery.

**Figure 4:**
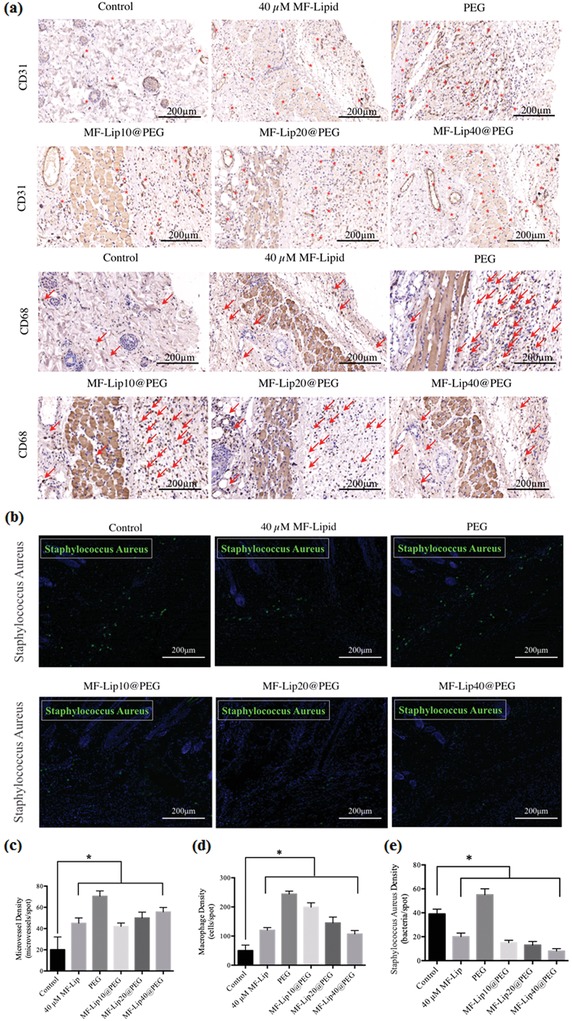
Effect of MF‐Lip@PEG on random‐pattern skin flap neovascularization, inflammation, and infection. Immunohistochemical images of flaps highlighting a) blood vessel CD31‐positive endothelial cells (red dots mark the microvessels) and CD68‐positive macrophage/monocytes (red arrows mark the cells). b) Immunofluorescence images of flaps highlighting *S. aureus* (green). The c) average microvessel densities, the d) average macrophage densities and b) the average bacterial densities in different groups. *p < 0.05.

In addition to angiogenesis, local inflammation after hydrogel treatment is also vital to skin flap regeneration. Immunohistochemical staining for CD68 was performed in order to assess the effect of MF‐Lip@PEG on skin flap inflammation during the regeneration process. As shown in Figure 4, the CD68 staining in the PEG group was significantly higher than that in the MF‐Lip, MF‐Lip@PEG and control groups. The average macrophage density was 50 ± 19 cells per spot in the control group and 244 ± 10 cells per spot in the PEG group. The average macrophage density was 120 ± 9 cells per spot in the 40 × 10^−6^ m MF‐Lip group. Additionally, CD68 staining decreased as the concentration of MF‐Lip@PEG increased. The average macrophage densities were 199 ± 15 cells per spot in the MF‐Lip10@PEG group; 145 ± 20 cells per spot in the MF‐Lip20@PEG group; and 107 ± 12 cells per spot in the MF‐Lip40@PEG group. The results showed that the MF‐Lip@PEG could alleviate the mild local inflammatory reaction.

Hydrogels, as foreign bodies, usually give rise to the mild local inflammatory reaction. This response could cause reactive neovascularization, which is beneficial for skin flap regeneration (Figure 4). However, excessive and persistent inflammation is not beneficial to flap recovery. However, the above results showed that the skin flap distal end survival rate was significantly increased in the PEG group compared to the control group. This finding indicates that PEG may cause mild inflammation in the flap tissue but does not affect the skin flap survival rate.

The MF‐Lip@PEG is dynamic coordinative hydrogel that is formed by four‐arm‐PEG‐SH and Ag^+^ Ag–S coordination. During hydrogel degradation, the MF‐Lip@PEG can slowly release Ag^+^ into the skin flap. To explore the antibacterial ability of the hydrogel, *Staphylococcus aureus* immunofluorescence staining was performed. As shown in Figure 4b, compared to the control (39 ± 4 bacteria per spot), 40 × 10^−6^ m MF‐Lip (20 ± 3 bacteria per spot) showed an outstanding antibacterial property. The *S. aureus* bacteria numbers could be further decreased by treatment with the MF‐Lip10@PEG (15 ± 2 bacteria per spot), MF‐Lip20@PEG (13 ± 3 bacteria per spot), and MF‐Lip40@PEG (8 ± 2 bacteria per spot). This result significantly demonstrates the effective antibacterial property of the MF‐Lip@PEG.

Bacterial infection is another leading cause of random skin flap necrosis, as flap tissues are especially vulnerable in an early stage hypoxic environment. Ag^+^ is a well‐known antibacterial agent and has long been used for targeted therapy of wound bacterial infections to promote wound healing.^37^, ^38^ However, excessively high concentrations of Ag^+^ irons in the early stage of wound healing can damage the tissue cells and induce innate cell apoptosis. The MF‐Lip@PEG in vitro release and degradation characteristics (Figure 1) indicate that dense network of the MF‐Lip@PEG could retard the drug release rate and ensure prolonged degradation during the endurance period. This behavior leads to constant but more favorable release concentrations of Ag^+^ for accelerating skin flap healing. The number of *S. aureus* bacteria in the PEG group was 55 ± 5 bacteria per spot, higher than that in the control (39 ± 4 bacteria per spot) group and the rest of the hydrogel treatment groups. This result suggests that PEG and the other hydrogels used for the skin flap are potential infection sources, which may induce local tissue inflammation (as observed in Figure 4a) and infections. However, the MF‐Lip@PEG could significantly reverse the flap bacterial number, which was in accordance with the reverse of the flap inflammation results observed upon treatment with the MF‐Lip@PEG (Figure 4a). From these results, it was observed that MF‐Lip@PEG possessed an outstanding antibacterial ability.

In summary, we have developed a hydrogel consisting of multifunctional four‐arm‐PEG crosslinked with Ag^+^ and loaded with MF‐Lip. The MF‐Lip@PEG was injectable and had self‐healing properties, low swelling percentages, a long endurance period, and excellent drug dispersibility and release characteristics for prolonged drug delivery. In addition, the hydrogel possessed the distinctive functions of the combined effects of PEG, Ag^+^ and MF‐Lip. The in vitro study results demonstrated that the MF‐Lip@PEG exhibited an outstanding proangiogenic effect and a strong protective effect on cells against hypoxia‐induced apoptosis through regulation of the Bax/Bcl‐2/caspase‐3 pathway. The in vivo study demonstrated that the MF‐Lip@PEG promoted skin flap angiogenesis, decreased skin flap inflammation, reduced skin flap infection, and significantly enhanced the skin flap survival rate. Overall, the injectable multifunctional MF‐Lip@PEG holds great promise for effectively matching the renewal of tissue regeneration.

## Conflict of Interest

The authors declare no conflict of interest.

## Supporting information

Supplementary
