# Supplementary material for: Self‐Healing and Injectable Hydrogel for Matching Skin Flap Regeneration
Source: Adv Sci (Weinh). 2018 Nov 16;6(3):1801555. doi: 10.1002/advs.201801555 (PMC6364594; doi:10.1002/advs.201801555)
Supplement: Supplementary file 1 — Supplementary [file ADVS-6-1801555-s001.pdf]

Copyright WILEY-VCH Verlag GmbH & Co. KGaA, 69469 Weinheim, Germany, 2018.

Supporting Information

for *Adv. Sci.,* DOI: 10.1002/advs.201801555

Self-Healing and Injectable Hydrogel for Matching Skin Flap Regeneration

Xiyuan Mao, Ruoyu Cheng, Hongbo Zhang, Jinhong Bae, Liying Cheng, Lu Zhang, Lianfu Deng, Wenguo Cui, Yuguang Zhang,* Hélder A. Santos,* and Xiaoming Sun**

Supporting Information

Self-healing and injectable hydrogel for matching skin flap regeneration

Xiyuan Mao[1], Ruoyu Cheng[1], Hongbo Zhang[1], Jinhong Bae, Liying Cheng, Lu Zhang, Lianfu Deng, Wenguo Cui[*], Yuguang Zhang[*], Hélder A. Santos *, Xiaoming Sun[*]

[1] X. Mao, R. Cheng and H. Zhang contributed equally to this work.

Dr. X. Mao, Dr. J. Bae, Dr. L. Cheng, Dr. X. Sun, Prof. L. Zhang, Prof. Y. Zhang
Department of Plastic and Reconstructive Surgery, Shanghai Ninth People's Hospital, Shanghai JiaoTong University School of Medicine, 639 Zhi Zao Ju Road, Shanghai 200011, P. R. China
E-mail: 817073@sh9hospital.org (X. Sun), Zhangyg18@126.com (Y. Zhang)

Dr. R. Cheng, Prof. L. Deng, Prof. W. Cui
Shanghai Key Laboratory for Prevention and Treatment of Bone and Joint Diseases, Shanghai Institute of Traumatology and Orthopaedics, Ruijin Hospital, Shanghai Jiao Tong University School of Medicine, 197 Ruijin 2nd Road, Shanghai 200025, P. R. China
E-mail: wgcui80@hotmail.com;

Prof. W. Cui
State Key Laboratory of Molecular Engineering of Polymers, Fudan University, No. 220 Handan Road, Shanghai 200433, China.

Prof. H. Zhang
Department of Pharmaceutical Sciences Laboratory and Turku Center for Biotechnology, Åbo Akademi University, Turku FI-20520, Finland

Prof. H. A. Santos,
Drug Research program, Division of Pharmaceutical Chemistry and Technology, Faculty of Pharmacy, University of Helsinki, Helsinki FI-00014, Finland.
Helsinki Institute of Life Science (HiLIFE), University of Helsinki, Helsinki FI-00014, Finland.
Email: helder.santos@helsinki.fi

Materials

Preparation of Mangiferin liposomes: thin-film dispersion method was utilized to prepare Mangiferin(MF)-loaded liposome(MF-Lip) as previously described. Specifically, materials containing phosphatidylcholine(PC)/ cholesterol(chol)/ MF (4:1:0.2 w/w) were dissolved in methanol with ultrasound to get uniform solution. Then the solution was transferred into rotary evaporator with the speed of 50 r/min at 40 °C to get a faveolate film in the bottom of flask, under the vacuum condition, any trance of organic solution was eliminated. Further, the film was hydrated with preheated phosphate buffered saline (PBS) at 40 °C with the speed of 50 r/min to get primary liposomes, then the solution was ultra-sonicated and extruded through the polycarbonate membrane filters with the pore size of 450 nm and 220 nm, respectively, to obtain homogeneous MF-Lip.

Characterization of Mangiferin liposomes: Physicochemical characterization of Mangiferin liposomes: the size distribution, zeta potential and polydispersity indexes of MF-Lip were explored by Nano Series Zen 4003 Zeta Sizer. Further, the morphological characters of these nanoparticles were observed by transmission electron microscopy (TEM) (FEI Tecnai G-20) and Scanning electron microscope (SEM) (HitachiSU5000), separately. To be more specific, the solution of MF-Lip was diluted to appropriate concentration, and then was stained with 2 % phosphotungstic acid to make the structure of liposomes was more easy to observe by TEM. Additionally, the liquid of MF-Lip was lyophilized under the protection of trehalose to get powder of MF-Lip used to explore the surface of these particles by SEM.

Drug release of Mangiferin liposomes in vitro: firstly, entrapment efficiency of MF-Lip was investigated as following description, 1 mL MF-Lip solution was put in the upper section

of the ultrafiltration device (Millipore, Mw: 3500), and then was centrifugalized with the speed of 5000 r/min for 5 mins. When the process was complete, 1 mL fresh PBS was added into the upper part of the device and the centrifugalizing procedure was repeated to separate any unloaded MF. Subsequently, the concentration of MF in the bottomed solution was examined by high-performance liquid chromatography (HPLC) system with a UV detector (ShimadzuLC-2010A). The analysis was performed with an ODS column (100-5 C18 column, 250 × 4.6 mm, 5.0 μm particle size, J&K Chemica LTD. Shanghai) at 35 °C, and the mobile phases were consisted of acetonitrile and 0.1 % methanoic acid (170:830 v/v). Flow rate was 1.0 mL min^{-1} and the detection wavelength was set at 260 nm. Further, the MF-Lip was sealed in a dialysis bag (MW = 3500 Da) and then was immersed into PBS with (10 mM, pH 7.4) at 37 °C with the shake at 100 r/min. At setted time points, all of these release medium was replaced by fresh PBS, and the collected solution was tested by HPLC-UV to determine the concentration of MF. The test conditions were similar with abovementioned details.

Preparation of hydrogels: 4 arm PEG-thiol was regarded as a fundamental material for fabricating the self-healing and injectable hydrogel in this research. Firstly, 4 arm PEG-thiol (50 mg, 10.0 μmol theoretical thiols) powder was weighted and dissolved in 250 μL deionized water (DI water) to prepare the solution A. Further, 25 μL $AgNO_3$ (0.1M) solution was diluted in 225 μL of deionized water to prepare solution B. Then solution A and B were mixed to generate transparent gel named PEG after a few seconds. Additionally, MF-Lip was utilized to replace DI water during the process of preparation of solution A and B, which aimed to fabricate MF-Lip loaded PEG hydrogels with different concentration of MF-Lip. Specifically, MF-Lip loaded PEG hydrogels which contain 4 mg MF, 2mg MF and 1 mg MF respectively, were named MF-Lip40@PEG, MF-Lip20@PEG and MF-Lip10@PEG,

separately. In contrast, MF-loaded PEG hydrogel containing 2 mg MF was named as MF-PEG.

Characterization of hydrogels: Morphological examination: the powder of 4 arm PEG-thiol was dissolved in MF-Lip to prepare the solution A. Then liquid A was mixed with solution B containing MF-Lip and $AgNO_3$ to fabricate the MF-Lip@PEG. In order to exhibit the injectable capability of MF-Lip@PEG, some rhodamine was added into the solution during the process of preparation to make hydrogels more apparent. Then these hydrogels were injected through 1 mL syringe (the diameter of needle is 0.5 mm), subsequently, these hydrogels were injected into water. In order to explore whether MF-Lip will make significant influences on the morphology of hydrogels, these hydrogels were lyophilized and then cut into slices, these samples were observed by SEM to explore the surface characters of hydrogels.

Physicochemical characterization and drug release behavior of hydrogels: to explore the self-healing property of hydrogel, strain-dependent oscillatory measurements were firstly conducted on hydrogel to determine the critical strain value required to disrupt the gel network and transition to a solution state. Further, step–strain measurements were established to investigate the self-healing property of hydrogel, specifically, at the first 100 secs, hydrogel was subjected to low strain (γ = 0.05 %) followed by high strain (γ = 500 %) for 50 secs. When the high strain was discontinued then a low magnitude strain (γ = 0.05%) was applied. Additionally, a hole was created in the center of hydrogel, 15 min later the appearance of hydrogel was recorded by a digital camera at macro to explore the process of self-healing.

MF-PEG, MF-Lip10@PEG, MF-Lip20@PEG and MF-Lip40@PEG hydrogel were freeze-dried to obtain the lyophilized samples. Subsequently, these hydrogels were weighted and immersed into DI water. At set time-point, these hydrogels were weighed and then datas were recorded, according to these datas, a curve was drawn to describe hydrogel's capability in water absorption. Those hydrogels for explore the degradation properties were soaked in DI water at 37 °C for 24 h to reach the equilibrium swelling state and then were weighed to record their initial masses. Further, hydrogels were kept in DI water at 37 °C with mild shaking, the remaining masses were regularly recorded at setted time points (days 1, 4, 8 and 12) to track the degradation kinetics.

The release behavior of these hydrogel were investigated by simulating drug release in vitro. To be more specific, these samples were immersed into PBS at 37 °C with the speed of 100 r/min in shaking bath. At setted time points, all release medium was replaced by fresh PBS, and these collected solution was analysed by HPLC-UV and related processing was similar as abovementioned details.

Reagents: CoCl2 · 6H2O(Sigma-Aldrich, USA) were dissolved in PBS at the concentration of 1M, solution were filter-sterilized (0.2 μM filter) and diluted to the desired concentration(50 μM~800 μM) before use.

Cell culture: HUVECs were purchased from the Shanghai Institute for Biological Sciences, Chinese Academy of Sciences (Shanghai, China). Cells were cultured in DMEM, high glucose (Gibco, USA) supplemented with 10% fetal bovine serum (Gibco, USA), 100U/ml penicillin, 100mg/ml streptomycin (Gibco, USA). Cells were incubated at 37°C with 5% CO_2.

MF-PEG leaching liquor: MF-PEG with liposomes carrying Mangiferin were soaked in PBS in 50 ml centrifuge tube for 24 h at room temperature, then the PBS were removed and fresh high glucose DMEM were added into the tube for another 48 h at room temperature. The leaching liquor went through 0.22 μM filters, and 10% fetal bovine serum (Gibco, USA), 100U/ml penicillin, 100mg/ml streptomycin (Gibco, USA) were added into the leaching liquor before cell culture.

Western blot analysis: The HUVECs subjected to different treatments were washed with PBS and homogenized with RIPA lysis buffer blended with protease and phosphatase inhibitors on ice for 30 mins, and shattered by ultrasound, then the cell extracts were centrifuged and the supernatant were collected. The supernatant total protein was quantified using a Branford protein assay kit (KeyGEN Biotech, Nanjing, China). Total protein was separated by SDS-PAGE and then transferred onto PVDF membranes, the membranes were blocked for 1h in 5% skim milk diluted with TBS containing 0.1% Tween-20(TBST), followed by incubation with primary antibodies overnight at 4°C. The membranes were washed with TBST and then incubated with horseradish peroxidase(HRP)-conjugated secondary antibody for 1 h at room temperature. The immune complex images were developed by ECL in the dark and were detected using a fluorescence imaging analysis system. The quantification of protein expression was performed via Image J software (National Institutes of Health).

Flow cytometric analysis for apoptosis: Cells were seeded in 6-well plate, 3×105 cells/well, after culture for 24h, cells were treated with $CoCl_2$ and different concentrations of Mangiferin for 24h. Then the cells apoptosis was examined according to the manufacturer's instructions using the FITC Annexin V Apoptosis Detection Kit(BD Biosciences, San Jose, CA, USA).

The fluorescence intensity was measured using a Becton-Dickinson FACS Caliber flow cytometer(BD Biosciences).

Animals: The animals were purchased from the Shanghai Ninth People's Hospital animal center, male Sprague Dawley rats of 4～6 weeks old were used in the study, animals were maintained under specific pathogen-free conditions and fed ad libitum. Animal experiments were according to the international ethics guidelines and the National Institutes of Health Guide concerning the Care and Use of Laboratory Animals.

Rat random-pattern skin flap model and treatment: The rats were randomly assigned to 5 groups: control group, PEG group, PEG with 10 μM Mangiferin group, PEG with 20 μM Mangiferin group and PEG with 40 μM Mangiferin group respectively. Under sterile conditions, the rats were anesthetized, a 1.5cm(width)×5cm(length) area was marked on the rats' dorsal which was the determined random-pattern flap area, the pedicel of the flap was in the tail end. A full-thick skin flap was elevated, the underneath soft tissue was completely cut off from the flap, and any axial blood vessel come into the flap from the pedicle would be cut off. The flap was carefully sutured back to its original position, 0.3ml PEG and Mangiferin solution was injected intradermally into each flap from the distal end to the pedicle end. And the rats were returned to their cages after recovering from anesthesia.

Macroscopic evaluation and histologic analysis: The animals were anaesthetized and the surviving and necrotic areas of the flaps were photographed with a digital camera at day 7 of treatment, the necrosis of the flaps were determined by the color, gross appearance, tissue texture and the moorFLPI blood flow imager using the laser speckle contrast technique to capture real time blood flow images. The necrosis areas length were quantified as the

percentage of the flap total length and the results were considered as percentage of skin flap necrosis. 1cm×1cm specimens were harvested from the necrosis and survival junction areas of the flaps for histological assessment. All tissue samples were fixed with 4% neutral formalin for 24 hours, then the specimens were embedded in paraffin and cross-sectioned into 5 μM slices. The slices were stained using heatoxylin-eosin for light microscopy.

Immunohistochemical analysis of CD31, CD68 and microvessel density detection: Immunohistochemiscal analysis was performed on 5 μM thick paraffin-embedded tissue sections. The sections were dewaxed and quenched the endogenous peroxidase activity with 3% hydrogen peroxide for 10 min, then blocked with the corresponding serum from a secondary antibody raised animal species for 1 h. The slices were incubated with the primary antibody (mouse anti-CD31 antibody,1:100; Abcam, USA; mouse anti-CD68 antibody,1:100; Abcam, USA), at 4 ˚C overnight, then added anti-rat secondary antibody at a dilution of 1:200 in PBS and incubated at room temperature for 30 min. After washed with PBS for several times, the signals on the tissue were revealed by incubating with DAB in PBS for 5~10 min, then hematoxylin counterstaining. The sections were observed under microscope. Microvascular density was evaluated at 100× magnification, from the most vascularized area three areas were selected, then the microvessels were counted from those three areas at 200× magnification. The average count of the three areas was considered as the microvascular density.

Immunofluorescence staining of staphylococcus aureus and bacteria density detection: Immunofluorescence analysis was performed on 5 μM thick paraffin-embedded tissue sections. The sections were dewaxed and quenched the endogenous peroxidase activity with 3% hydrogen peroxide for 10 min, then blocked with the blocking buffer for 1 hour. Slices were incubated with the primary antibody (mouse anti-staphylococcus aureus antibody,1:100;

Abcam, USA) at 4 °C overnight, then incubated in secondary antibody diluted in antibody dilution buffer at room temperature for 1 hour in dark. After washed with PBS for several times, coverslip slides with anti-fade reagent with DAPI. The sections were observed under fluorescence microscope. Staphylococcus aureus bacteria density was evaluated at 100× magnification, from the most cell dense area three areas were selected, then the cells were counted from those three areas at 200× magnification. The average count of the three areas was considered as the staphylococcus aureus bacteria density.

Statistical analysis: All the experiments were preformed in 3 replicates. Values are expressed as the means ± SD, and the difference between groups was analyzed by one-way ANOVA with Tukey's and Newman Keuls post tests. Statistical analysis was performed by SPSS (IBM Corp., Armonk, NY, USA). P-value<0.05 were considered statistically significant difference.

Figures

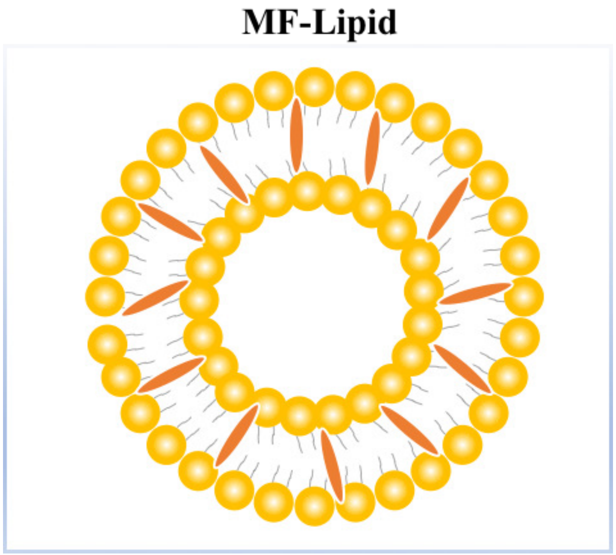

Figure S1. The diagram of MF-Lip .

TEM

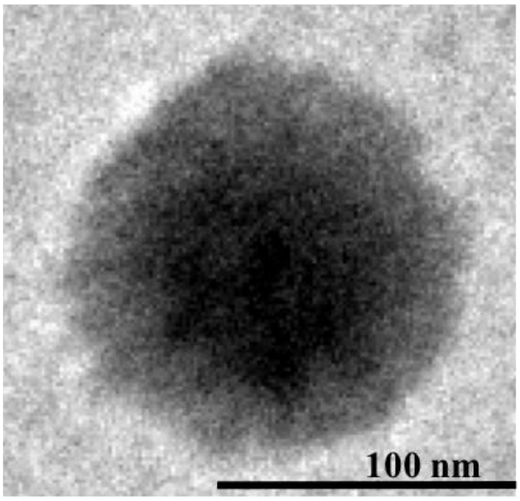

Figure S2. The Morphological examination of MF-Lip. TEM image of MF-Lip.

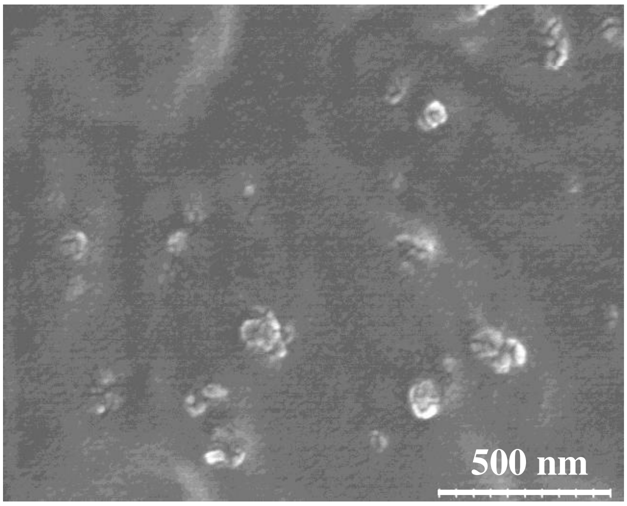

Figure S3. The Morphological examination of MF-Lip. SEM photograph of MF-Lip.

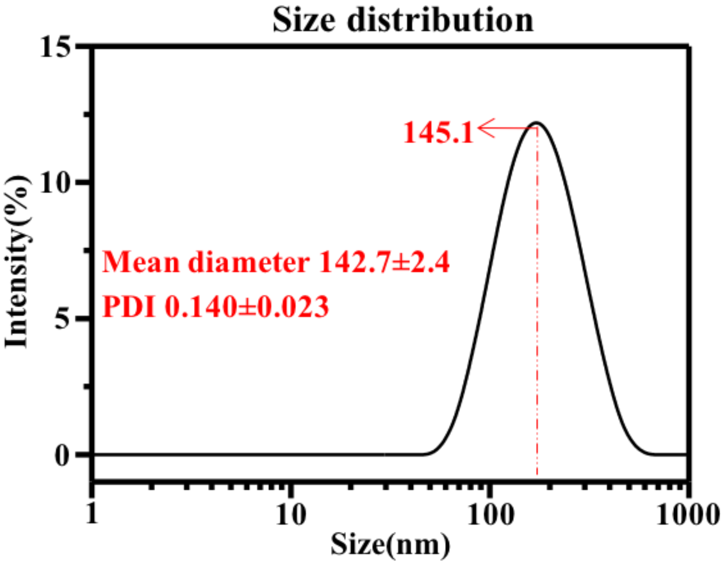

Figure S4. Related characters about MF-Lip. The size distribution.

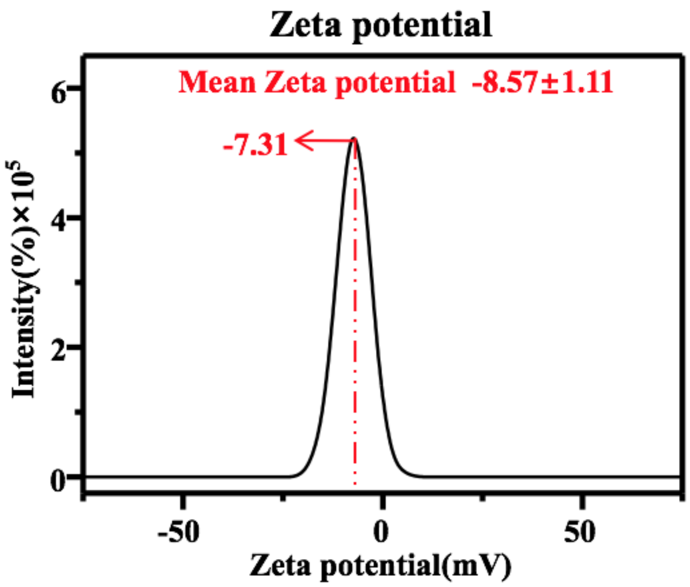

Figure S5, Related characters about MF-Lip. The zeta potential distribution.

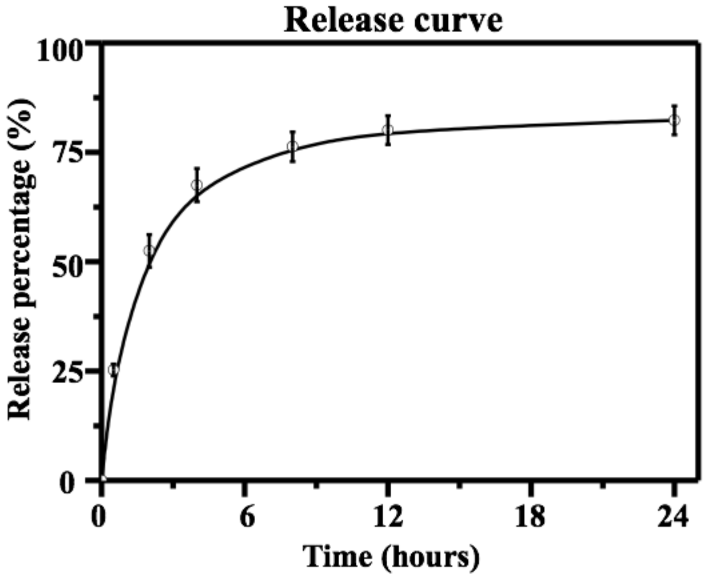

Figure S6. Related characters about MF-Lip. The drug release curve in vitro.

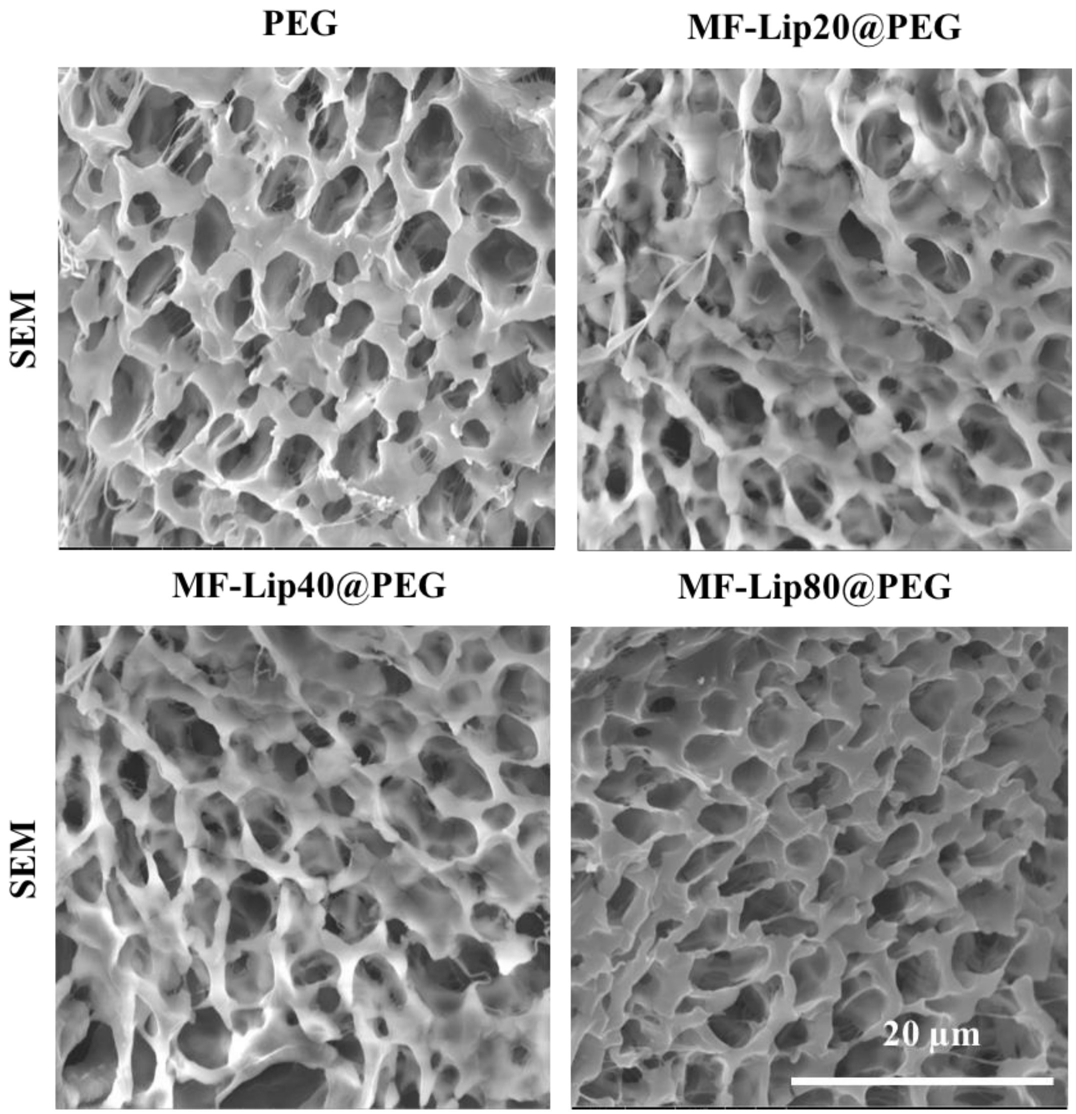

Figure S7. The Morphological examination of hydrogels. SEM results at low magnification

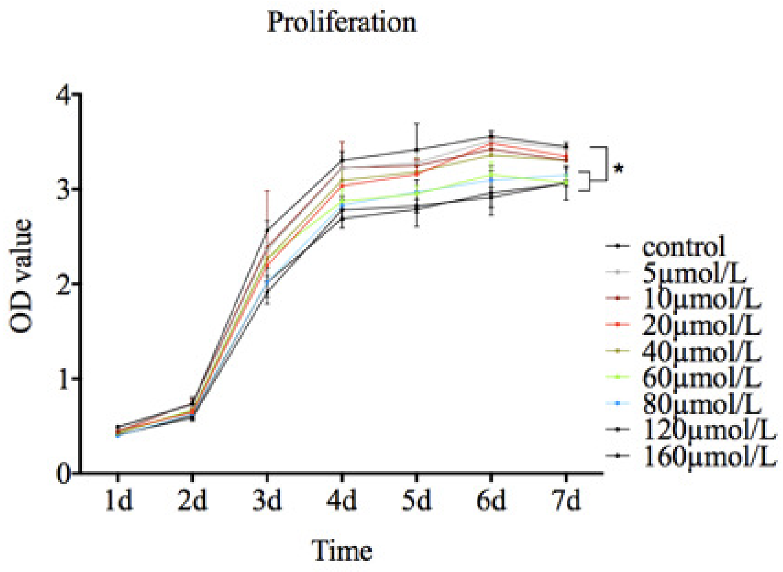

Figure S8. Cell viability of HUVECs in DMEM with different concentrations of Mangiferin detected by CCK-8 assay, cell viability was tested everyday for 7 days in a row. *p<0.05.

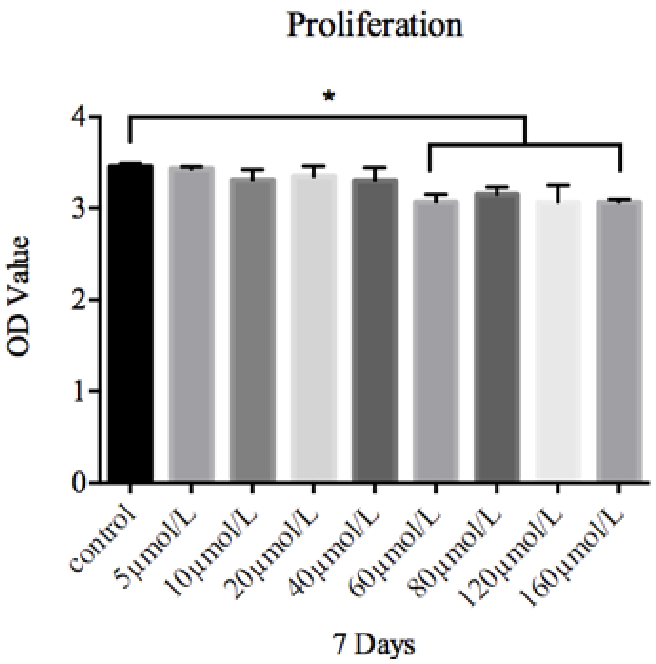

Figure S9. Cell viability of HUVECs in DMEM with different concentrations of Mangiferin on day 7, there were significantly difference in cell viability of groups with mangiferin concentration above 60 μM. *p<0.05.

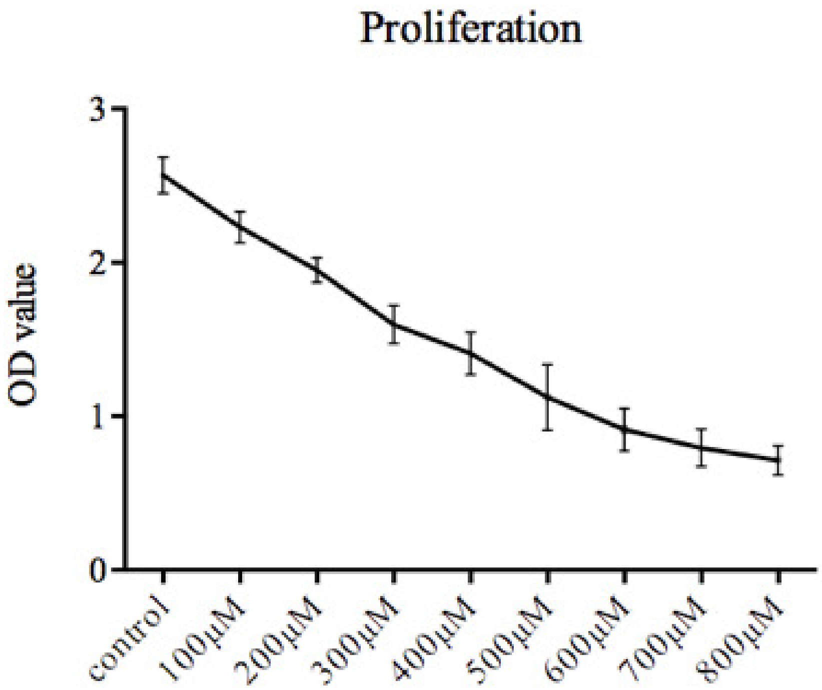

Figure S10. Cell viability of HUVECs in DMEM with different concentrations of $CoCl_2$ after 24 h was detected by CCK-8 assay, the cell viability of the 400 μM group was about 50%. Therefore, 400 μM $CoCl_2$ was chosen for the following investigation.

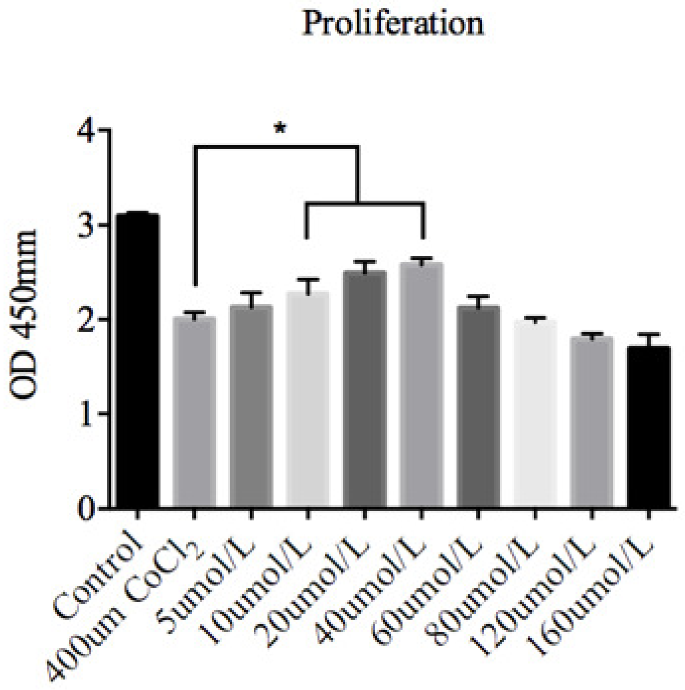

Figure S11. Cell viability of HUVECs in DMEM with different concentrations of mangiferin and 400 μM $CoCl_2$ after 24 h was detected by CCK-8 assay. Cell proliferation was significantly improved by Mangiferin under the concentration of 40 μM in a dose-depended manner, while Mangiferin above the concentration of 60 μM showed no protective effect on the cells. *$p<0.05$.

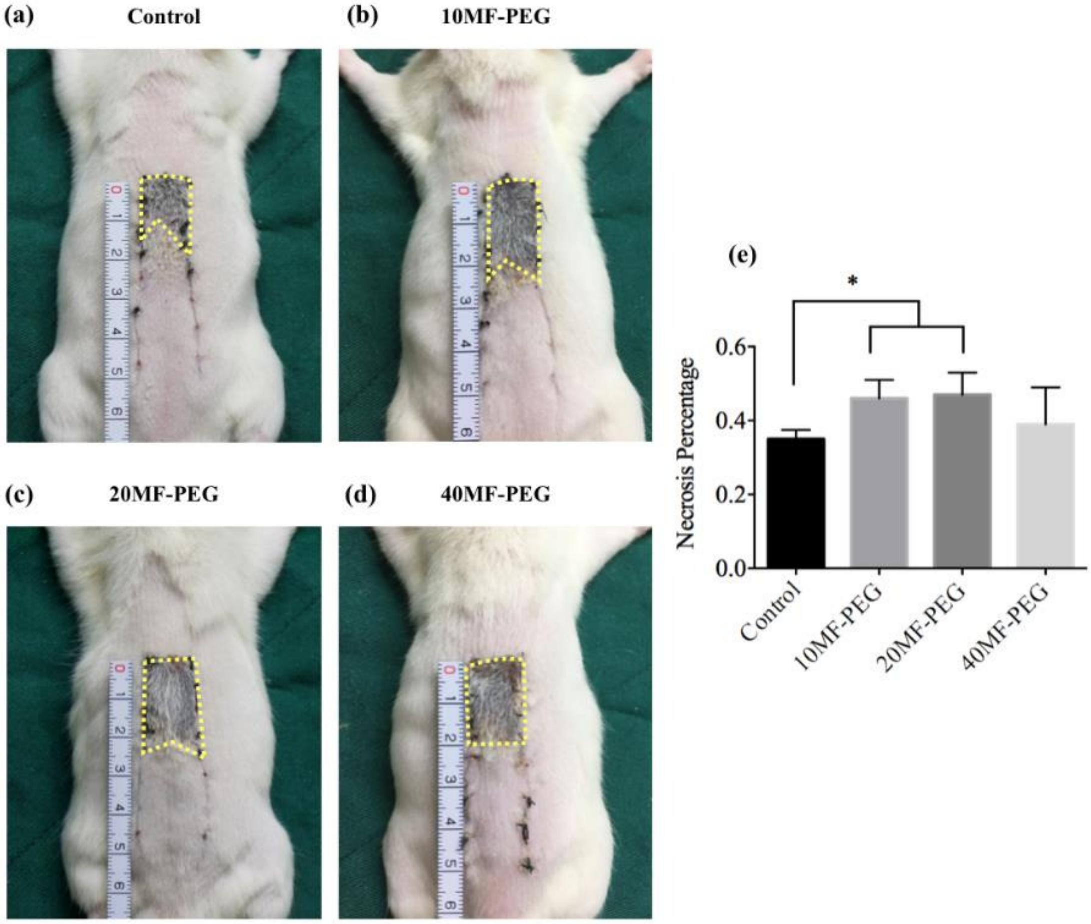

Figure S12. The MF-PEG on random skin flap survival ratio. (a) Control. (b) 10 MF-PEG. (c) 20 MF-PEG. (d) 40 MF-PEG. (e) The skin flap necrosis percentage in the corresponding groups at day 7 after operation. *$p < 0.05$. The necrosis areas were marked with yellow frame.
